# Glioblastoma surgery related emotion recognition deficits are associated with right cerebral hemisphere tract changes

**DOI:** 10.1093/braincomms/fcaa169

**Published:** 2020-10-12

**Authors:** Rohitashwa Sinha, Aicha B C Dijkshoorn, Chao Li, Tom Manly, Stephen J Price

**Affiliations:** 1 Cambridge Brain Tumour Imaging Laboratory, Department of Clinical Neurosciences, University of Cambridge, Cambridge, UK; 2 MRC Cognition and Brain Sciences Unit, University of Cambridge, Cambridge, CB2 7EF, UK

**Keywords:** glioblastoma, surgery, cognition, function, registration

## Abstract

Patients with glioblastoma face abysmal overall survival, cognitive deficits, poor quality of life and limitations to social participation; partly attributable to surgery. Emotion recognition deficits mediated by pathophysiological mechanisms in the right inferior fronto-occipital fasciculus and right inferior longitudinal fasciculus have been demonstrated in traumatic brain injury and dementia, with negative associations for social participation. We hypothesize similar mechanisms occur in patients undergoing resection surgery for glioblastoma. Here, we apply tract-based spatial statistics using a combination of automated image registration methods alongside cognitive testing before and after surgery. In this prospective, longitudinal, observational study of 15 patients, surgery is associated with an increase in emotion recognition deficits (*P *=* *0.009) and this is correlated with decreases in fractional anisotropy in the inferior longitudinal fasciculus, inferior fronto-occipital fasciculus, anterior thalamic radiation and uncinate fasciculus; all in the right hemisphere (*P *=* *0.014). Methodologically, the combination of registration steps used demonstrate that tract-based spatial statistics can be applied in the context of large, scan distorting lesions such as glioblastoma. These results can inform clinical consultations with patients undergoing surgery, support consideration for social cognition rehabilitation and are consistent with theoretical mechanisms that implicate these tracts in emotion recognition deficits across different diseases.

## Teaser

Sinha *et al.* report that glioblastoma surgery is associated with emotion recognition deficits. Using automated image registrations for large lesion distortion and tract-based spatial statistics, this deficit is shown to be associated with changes in the same right hemisphere white matter tracts as seen in other diseases.

## Graphical Abstract

**Graphical Abstract:**
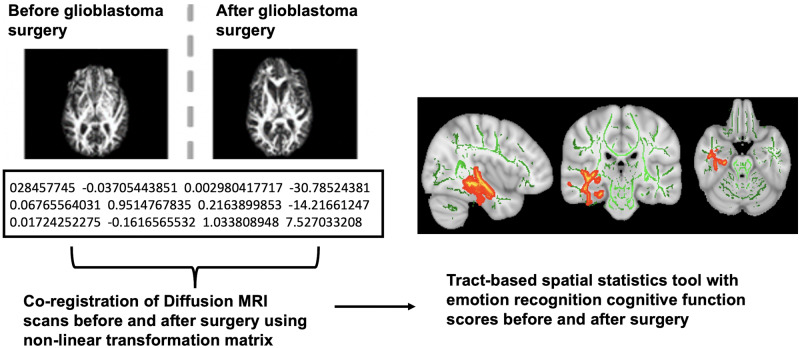

## Introduction

Glioblastoma is the most common malignant primary brain cancer. It has a median survival ranging from 14 to 17 months after maximal therapy with surgical resection, radiotherapy and Temozolomide chemotherapy (Stupp *et al.*, 2015). Cognitive deficits and associated poor quality of life further exacerbate the burden patients face, partially attributable to the current treatments (Giovagnoli, 2005). With no imminent cures on the horizon, one important area where patients may benefit is by understanding and minimizing treatment-related cognitive dysfunction (Peters *et al.*, 2016). Even if survival were to increase, cognitive dysfunction would still be pertinent.

According to a published report surveying their lived experience, patients with brain tumours withdraw from social participation following treatment (Brain Tumour Charity, 2015). This is similar to patients with traumatic brain injury and dementia where emotion recognition deficits are associated with negative interpersonal interactions, increased caregiver burden and poor quality of life (Snowden *et al.*, 2008; Genova *et al.*, 2015). Data from diffusion tensor imaging (DTI) studies *using tract-based spatial statistics* (TBSS) of patients with traumatic brain injury, multiple sclerosis, Parkinson’s disease and schizophrenia converge on fractional anisotropy (FA) abnormalities in the Inferior longitudinal fasciculus (ILF) and inferior fronto-occipital fasciculus (IFOF) being implicated in the pathophysiological mechanism of emotion recognition deficits (Baggio *et al.*, 2012; Mike *et al.*, 2013; Genova *et al.*, 2015; Zhao *et al.*, 2017).

Emotion recognition deficits also occur in patients with brain tumours undergoing surgery (Campanella *et al.*, 2015). However, this has not specifically been studied in the context of glioblastoma. This cognitive ability is not covered by standardized neuropsychological testing (Wefel *et al.*, 2011).

Glioblastoma preferentially infiltrates along white matter (WM) tracts (Scherer, 1940). As surgeons aim to remove as much tumour as possible for increasing survival, surgery for glioblastoma carries additional risk of disrupting key WM tracts and worsening social cognition functions such as emotion recognition.

We aim to identify WM tracts associated with emotion recognition deficits in patients with glioblastoma, from before to after resection surgery; when these structures and functions are at risk. We hypothesize that FA reductions in the ILF and IFOF correlate with emotion recognition deficits, as with other pathologies.

## Materials and methods

### Study design

We conducted a prospective, longitudinal, observational study. To detect a longitudinal emotional recognition deficit in glioblastoma patients from before to after surgery at 80% power and 5% significance, we used the healthy control mean of 14.21 (standard deviation, SD* *=* *2.51), deficit cut-off score of 11 (at 2 SD) and effect size of 1.3; comparable to other clinical versus healthy population cognitive screening instruments (Larner, 2014). The sample size calculation stipulated eight paired sets of patient data, adjusted for T-distribution. All longitudinal data were analysed using paired sample tests to avoid pseudoreplication.

### Participants

We recruited adult patients admitted to Addenbrooke’s hospital between May 2017 and January 2019, in approved studies investigating longitudinal DTI and cognition in patients with glioblastoma (Essex and Harrow Research Ethics Committees, 16/EE/0467 and 18/LO/0491, respectively). Written informed consent was obtained from all patients. All procedures were in accordance with the Declaration of Helsinki. Inclusion criteria were (i) diagnosis of glioblastoma, (ii) intended surgical removal of at least 90% of the enhancing tumour, (iii) suitable for subsequent radiotherapy (60 Gray) with concomitant Temozolomide, (iv) World Health Organization performance status of zero or one and (v) intact capacity for longitudinal cognitive testing before and after surgery. All patients underwent preoperative DTI-scan in the week before surgery and a second postoperative DTI-scan up to 4 weeks after surgery, but prior to radiotherapy initiation.

### Neuropsychological assessment

These were performed using tests in the tablet-based screening battery OCS-Bridge (https://ocs-bridge.com/) during ‘presurgical-assessment’ visits and postoperatively on the day of hospital discharge or before the first clinic review thereafter. Emotion recognition was evaluated by the Affective Facial Expression Test in which patients were asked to select the correct emotional expression for 18 faces, displaying 6 basic emotions (happiness, sadness, anger, surprise, fear and disgust). These were static photograph stimuli rather than video footage. Contrasting test conditions were used to assess bias from co-existing visual perception deficits. Specifically, the ‘Faces and Beach Huts Test’ for neutral face and object recognition deficits and Line Bisection Test for field bias as seen in neglect. Anxiety and mood was assessed with Generalised Anxiety Disorder-7 and Patient Health Questionnaire-9 (Kroenke *et al.*, 2001; Spitzer *et al.*, 2006). Postoperative testing used parallel tests in OCS-Bridge and scores were corrected against normative control data for reliable change.

### MRI acquisition

Data acquisition was performed before and after surgery with a 3T Siemens MR scanner with 12-channel head coil. We acquired 64-direction DTI scans (*b* = 0 s/mm^2^ and *b* = 1000 s/mm^2^), T_2_-weighted, pre-contrast and post-contrast T_1_-weighted sequences (see Supplementary material 1).

### Image pre-processing

**Figure 1:**
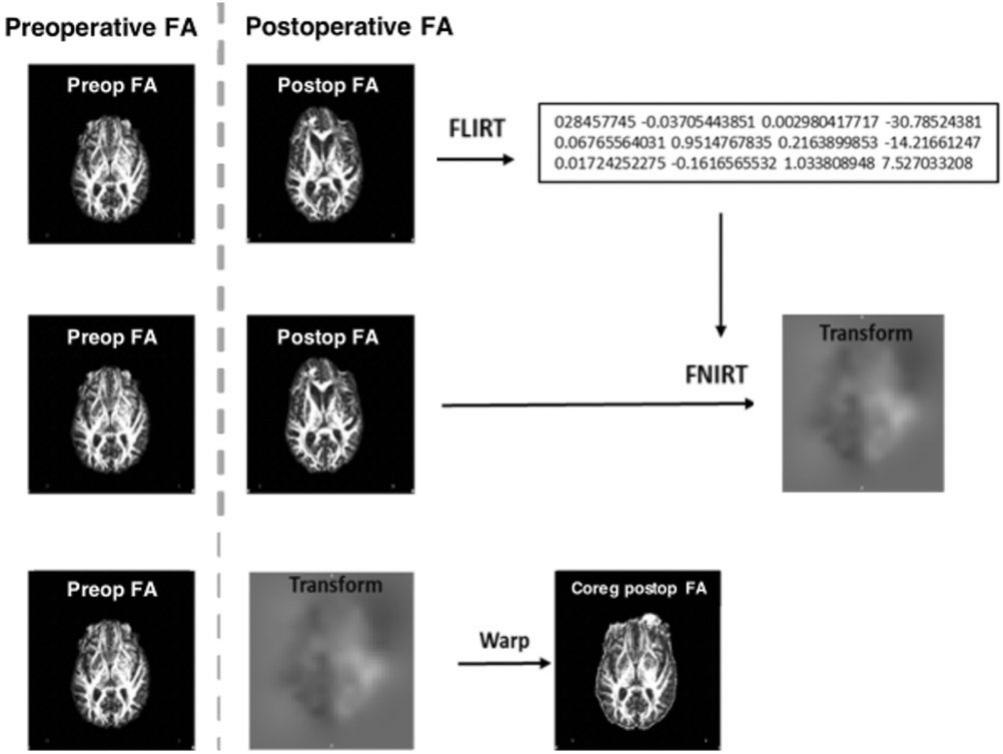
C**o-registration diagram with use of transformation matrix.** Overview of the processing steps resulting in the co-registration of the follow-up DTI image with the preoperative DTI image. First, linear co-registration is applied (FLIRT). Next, the generated matrix is implemented into the non-linear co-registration step (FNIRT) to create a non-linear matrix (Transform). The transformation matrix will determine the amount of warping that is needed to co-register the follow-up image to the preoperative image for each subject.

DTI data was processed using FSL version 5.0.11 (Smith *et al.*, 2004). (i) Subject motion and eddy-current distortions were corrected with affine registration to the b0 image. (ii) Brain extraction was performed with the automated Brain Extraction Tool from the non-diffusion weighted b0 image. (iii) DTIFIT was used to apply diffusion tensor models at each voxel and FA maps were calculated. All imaging passed visual inspection to ensure general data quality. (iv) Each subject’s postoperative FA map was co-registered to their preoperative FA map to improve within-subject alignment of the central FA tracts. To achieve improved alignment (Fig. 1), we applied linear registration (FLIRT) to permit initial affine registration and to generate an estimated transformation matrix (Jenkinson and Smith, 2001). This transformation matrix was applied to guide the non-linear registration (FNIRT) in normal FMRIB58_FA standard space (Woolrich *et al.*, 2009). These automated co-registration steps have been adapted from a validated method applied previously for patients with glioblastoma before and after surgery (Van der Hoorn *et al.*, 2016). To correct for tumour lesion effect, volume was calculated from the segmented tumour region based on the multimodal MRI sequences using BraTumIA tool version 2.0 (software.istb.unibe.ch/registration/bratumia/) (Supplementary material 2).

### Tract-based spatial statistical analysis

The co-registered FA images were analysed for voxel-wise within-subjects × cross-subjects’ statistics using TBSS, version 1.2, in FSL 5.0.11. Detailed description of TBSS steps were reported (Smith *et al.*, 2006). Briefly: (i) The non-linear registration tool FNIRT transforms all co-registered FA images into higher-resolution 1 mm × 1 mm × 1 mm FMRIB58_FA common space (Woolrich *et al.*, 2009). (ii) The mean FA image across all subjects was created and skeletonized at the recommended threshold of 0.2 (Smith *et al.*, 2006). The mean FA skeleton represents the position of central FA tracts common to all subjects, for further consideration. All participants’ data passed visual checks that the threshold mean FA skeleton overlay central tracts for each participant. (iii) These aligned FA images were projected onto the 0.2 threshold mean FA skeleton. (iv) The resulting data were fed into within-subject × cross-subject voxel-wise statistical analysis at each voxel on the common skeleton.

### Data availability

The cognitive task data and scripts for this study are available online (Supplementary Table 3 and material 3). The neuroimaging data will be available via the Cancer Research UK Imaging repository following an embargo period.

## Results

### Demographics and clinical characteristics

Twenty-three patients were prospectively included at baseline. Eight patients were excluded to minimize bias: four patients became ineligible from post-surgical strokes, two patients had progressive disease deterioration following surgery, one patient had adjuvant treatment at another hospital hence follow-up was incomplete and one patient could not attend follow-up cognitive assessment. Our final sample size for longitudinal analysis comprised 15 patients (35% drop-out rate), for whom datasets were complete.

At baseline, the excluded and included patients did not differ on any demographic or tumour characteristic measurements. The excluded patients had a mild leftward deviation on the line bisection task (M = −3.4 mm, SD* *=* *3.8 mm) compared with the participating patients (M* *=* *1.4, SD* *=* *3.1), independent sample *t*-test (*t*(22) = −3.3, *P* = 0.003). There was no difference on the remaining cognitive measurements (Supplementary Table 1).

For demographic and clinical characteristics of the included patients (see Table 1). Preoperative cognitive assessment was performed (median) 3 days (range: 0–10) before surgery. Follow-up assessment was (median) 3 days after surgery (range: 2–10). Preoperative DTI scans were obtained (median) 5 days (range: 0–17) before surgery and follow-up scans (median) 21 days (range: 13–71) after surgery, arranged for patient convenience during routine clinical appointments.

**Table 1:** Demographic and clinical details

Sex	Age	Years of education	Tumour lateralization	**Tumour location** ^a^	Lesion volume (cm^3^)	IDH1	EOR > 90%	**Preop cog deficits** ^b^	**Postop cog deficits** ^b^	**Highest dose of dexamethasone (mg)** ^c^
M	63	14	Right	Temporal	37	Wild type	Yes	0/15	4/15	8
M	68	16	Left	Frontal	146	Wild type	Yes	1/15	1/15	16
M	61	31	Right	Occipital	26^d^	Wild type	Yes	0/15	3/15	16
F	65	14	Right	Temporal	59	Wild type	Yes	3/15	4/15	16
F	53	20	Left	Frontal	44	Wild type	Yes	0/15	1/15	8
M	55	16	Left	Temporal	45^e^	Wild type	Yes	1/15	1/15	0
M	58	18	Right	Occipital	105^e^	Wild type	Yes	6/15	7/15	16
M	50	11	Left	Temporal	54^e^	Wild type	Yes	0/15	5/15	16
M	57	11	Right	Temporal	125	Wild type	Yes	1/15	0/15	16
M	61	17	Right	Occipital	59	Wild type	Yes	2/15	6/15	8
M	28	16	Left	Frontal	62	Mutant	Yes	0/15	0/15	2
F	73	13	Right	Occipital	80	Wild type	Yes	5/15	5/15	2
F	58	21	Left	Temporal	36	Wild type	Yes	1/15	1/15	2
F	55	21	Left	Frontal	89	Wild type	Yes	3/15	8/15	16
M	59	20	Left	Temporal	69	Wild type	Yes	3/15	3/15	8

a As verified by the clinical report of a Consultant Neuroradiologist.

b Calculated as the number of (sub) domains from the OCS-BRIDGE tool that were (labelled as impaired, based on any scores lower than the fifth percentile cut-off point in three hundred sex, age and education matched controls.

c Electronic medication chart records held dosages of medication prescriptions rather than number of doses, from which the highest dose of Dexamethasone steroid medication was extracted as a surrogate measure of extent of treatment with steroid.

d Due to observed mis-segmentations of areas around the eye cavity as tumour tissue, we performed skull stripping with the Brain Extraction Tool in FSL.

e Due to observed segmentation errors the values were replaced with the groups’ mean tumour lesion volume (normally distributed) which corresponded to 69 cm^3^.

Cog, Cognitive; EOR, extent of resection; GB, glioblastoma; IDH1, isocitrate dehydrogenase 1 enzyme; Postop, postoperative; Preop, preoperative.

### Emotion recognition and visual perception task performance

Table 2 contains longitudinal cognitive performance details and Supplementary Table 2 contains raw cognitive task data scores. Before surgery, none of the patients with an emotion recognition deficit also had deficits in the other tests. After surgery, two patients had co-existing deficits in both emotion and face recognition tasks. In addition, one patient had deficits in line bisection, face and emotion recognition tasks. After surgery, patients scored significantly lower on Generalised Anxiety Disorder-7 anxiety (*t*(14) = 2.9, *P* = 0.012, 95% CI [1.05, 6.95]), but not Patient Health Questionnaire-9 depression measures (*t*(14) = 1.8, *P* = 0.094, 95% CI [−0.33, 3.80]), compared to before surgery. However, there was no relationship between the longitudinal changes in emotion recognition and anxiety scores (*bivariate Pearson correlation test*, *r* = −0.10, *P* = 0.717, two-tailed). For contextual comparison to normative data, healthy controls emotion recognition mean score was 14.21 (SD* *=* *1.82) on the first test and was 13.38 (SD* *=* *2.01) on the parallel re-test (Supplementary Table 3).

**Table 2:** Longitudinal cognitive test performance data

Cognitive test	Outcome type	Before surgery	After surgery	**Longitudinal difference** ^a^
Emotion recognition	Mean score	12.1 (SD* *=* *2.5)	10.8 (SD* *=* *2.7)	*t*(14) = 3.0, *P* = 0.009, 95% CI [0.39, 2.32]
	Number with deficit	3/15 (20%)	7/15 (47%)	
Face recognition	Mean score	5.4 (SD* *=* *0.7)	4.8 (SD* *=* *0.8)	*t*(14) = 1.9, *P* = 0.082, 95% CI [−0.09, 1.29]
	Number with deficit	2/15 (13%)	6/15 (40%)	
Object recognition	Mean score	4.9 (SD* *=* *1.2)	4.7 (SD* *=* *0.6)	*t*(14) = 0.9, *P* = 0.364, 95% CI [−0.34, 0.88]
	Number with deficit	2/15 (13%)	0/15 (0%)	
Line bisection	Mean deviation (mm)	1.4 (SD* *=* *3.1)	3.1 (SD* *=* *6.4)	*t*(14) = −1.4, *P* = 0.188, 95% CI [−4.54, 0.98]
	Number with deficit	1/15 (7%)	4/15 (27%)	

a Paired sample *t*-test. Data shown as *t*(degrees of freedom) = *t*-statistic, *P*-value, 95% confidence interval range.

### Longitudinal changes in FA tracts with cognitive scores after tumour volume correction

**Figure 2:**
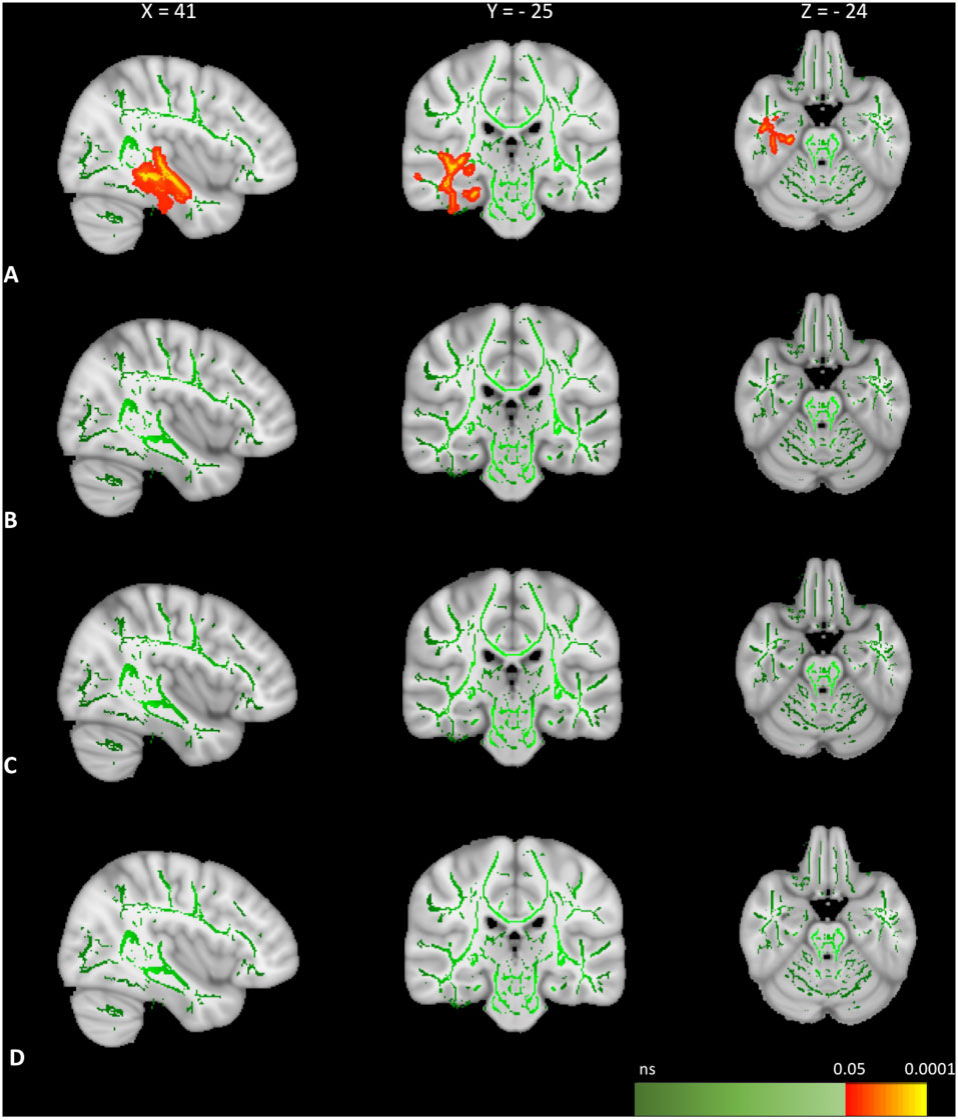
**TBSS correlation analysis FA-tract changes and cognitive test performance.** Correlation analysis between longitudinal changes on performance on social cognition and visual perception tasks and longitudinal FA-tract changes after correction for preoperative tumour lesion volume. (**A**) There is a significant positive correlation (*P* < 0.05) between decreased performance on the emotion recognition task and FA tract deterioration (TFCE-corrected) in the right ILF (displayed in sagittal plane) and in the right IFOF (displayed in coronal plane). The right Cingulum bundle with anterior thalamic radiation and uncinate fasciculus is displayed in the axial plane. There is no significant correlation (*P* > 0.05) between deterioration in any of the FA tracts and decreased performance on the neutral face recognition task (**B**) object recognition task (**C**) or line bisection task (**D**).

TBSS voxel-wise linear modelling revealed significant positive correlation between reduced performance on the emotion recognition task and reduced FA tract values in the right ILF, right IFOF, right uncinate fasciculus) and right Anterior Thalamic Radiation (*P* = 0.014, Max MNI coordinates : [*X*  =  38, *Y* = −10, Z = −16]) (see Supplementary Table 4 for FA-tract coordinates). There were no correlations between longitudinal *increase* in FA tract values and longitudinal changes in emotion recognition performance. Voxel-wise correlation analysis results are shown in Fig. 2. The significance image map with multiple equidistant slices from multiple planes is provided in Supplementary Fig. 1. No significant relationship was found between longitudinal changes in FA tract values and longitudinal changes in performance on the neutral face recognition, the object recognition or the line bisection task (Fig. 2).

## Discussion

This is the first TBSS study to investigate the longitudinal relationship between changes in emotion recognition and changes in FA tract values in patients with glioblastoma undergoing resection surgery. As hypothesized, our data revealed that reduced FA in the right IFOF and the right ILF are associated with reduced ability to accurately recognize emotional expressions after surgery. In addition, our findings also implicated the right uncinate fasciculus and right Anterior Thalamic Radiation tracts. Our use of clinical DTI-scan protocols from a conventional 3T scanner (widely available in most hospitals) coupled with freely available and automated methods promote observer independence and research reproducibility.

There was no correlation between changes in FA and changes in performance on contrast condition data for neutral face recognition, object recognition or visual field bias. Neither did we find any correlation between performance on the emotion recognition task and any of the visual perception tasks on a group level. Importantly, there was no relationship between cognitive test performance data and areas of surgery-associated FA increase. This suggests unidirectionality of the effect without spurious findings.

We show that emotion recognition impairment is already present in a subgroup of patients before surgery and becomes more frequent after surgery, when patients are first discharged from the hospital. This is when they receive information about their poor prognosis and cannot return to work or driving. All of which may impact the social isolation reported by this patient group (Brain Tumour Charity, 2015).

The relationship between reduced FA values in the ILF and IFOF and deficits in emotion recognition deficits is in accordance with reports from other clinical populations (Philippi *et al.*, 2009; Baggio *et al.*, 2012; Mike *et al.*, 2013; Genova *et al.*, 2015; Zhao *et al.*, 2017) and healthy controls (Philippi *et al.*, 2009; Genova *et al.*, 2015; Unger *et al.*, 2016). These findings also support the hodological anatomical framework for disordered visual perception of facial emotion (Ffytche *et al.*, 2010), although assessing the potential ‘hypoconnection’ associated features of hypo-emotionality and de-realization were outwith the scope of the current study.

Interestingly, we found that emotion-recognition task performance was associated with the FA overlying limbic tracts. The uncinate fasciculus has been associated with social-emotional processes as well as memory (Von Der Heide *et al.*, 2013). The Anterior Thalamic Radiation has been implicated in affective processing and emotion regulation (Downey *et al.*, 2015). These limbic tracts are often seen disrupted in diseases with socio-affective component (Wang *et al.*, 2018). From theoretical viewpoints, our data support the commonality of WM tract-mediated mechanisms found with emotion recognition deficits across multiple pathologies, now including glioblastoma.

At group level, all emotion recognition-FA correlates were found in the right hemisphere, despite half the sample having left hemisphere lesions visible on MRI whilst all being right-hand dominant. The right hemispheric lateralization of emotion recognition is congruent with the prominent role of the right hemisphere in face recognition (Tavor *et al.*, 2014; Unger *et al.*, 2016) and perception of emotional expressions (Adolphs, 2002).

The observed FA changes may be attributable to surgery-associated cellular injury cascades or physiological shifts in intracranial water content, such as oedema. We applied 0.2 threshold to the FA skeleton to account for cerebrospinal fluid partial volume effects (Smith *et al.*, 2006) and possibly oedema-related partial volume effects. The FA decreases may represent changes in global connectivity from the disease (Stoecklein *et al.*, 2020) or local WM integrity from injury during tumour resection. Myelin sheets and axonal membranes restrict diffusion perpendicular to their axis, hence isotropic water diffusion (lower FA values) could reflect decreased axonal membrane integrity (Beaulieu, 2002); potentially exacerbated by surgery.

The effect of steroid medication on emotion recognition function may also contribute to the deterioration seen in this cohort. At our unit, Dexamethasone steroid is commonly used to alleviate tumour mass effect and symptoms prior to surgery, with a weaning regimen postoperatively. We were able to obtain a surrogate measure in the highest dose of Dexamethasone used peri-operatively from the electronic medication charts (Table 1). *Post hoc* analysis of these dosages in the cohort revealed no significant differences when comparing participants with left versus right hemisphere surgery or when comparing participants who with emotion recognition deficits versus those without prior to surgery (Wilcoxon tests with *W *=* *36, *P* = 0.35 and *W *=* *30.5, *P* = 0.51, respectively). However, these data may lack the granularity that total doses and timings could provide as an explanatory variable in this context. Such granularity, whilst outwith the scope of this study, has previously shown that the variability in circadian versus ultradian pulsatility of steroid administration affects emotion recognition function using the same total daily dose (Kalafatakis *et al.*, 2018).

For clinical utility, our findings can inform patient consultations about possible surgery related deteriorations in emotion recognition ability. Since emotion recognition deficits are proposed to cause withdrawal from social events and increased caregiver burden (Snowden *et al.*, 2008; Genova *et al.*, 2015), this study supports the consideration of social cognition specific rehabilitation for this patient group to maintain quality of life. From a research perspective, our combination of FSL co-registration methods demonstrate a useful advance in successfully applying TBSS in patient groups with large, scan-distorting lesions such as glioblastoma.

## Limitations

Our sample size did not have the statistical power to separately analyse the effect of lesion location/hemisphere or to correct for variance in visual cognitive abilities, as the contrast test conditions included used less trials than the emotion recognition task (6 versus 18). Our cohort was biased by having more patients with occipital lobe-based tumours in the right hemisphere whilst the patients with frontal lobe-based tumours were all in the left hemisphere. Hence surgery involving occipito-temporal visual and face processing regions on the right may underpin the mechanism for our findings, but larger studies with balanced sub-groups for lobar location of the lesions may better support hemispheric differences.

Second, the cognitive tests using static photograph images do not equate to experience of daily problems. Multimodal measurements including video stimuli and outcomes at the level of social participation are needed to bridge this gap. Third, the quantification of any variance between operations was beyond the scope of this study but would be important to identify modifiable surgical factors to protect cognitive function. Fourth, the timing of postoperative cognitive tests did not exactly match the postoperative DTI scan acquisition (median 3 days versus 21 days postoperatively) to facilitate convenience for patients attending routine follow-up appointments. Hence, we have assumed that functional deficits found postoperatively persist for ∼18 days to relate to the structural imaging findings. A *post hoc* Fisher’s exact test using follow up data available for 13/15 participants supports this assumption. It showed no difference between their emotion recognition deficits (*P* = 0.23) when assessed early after surgery (median 3 days) and just before radiotherapy (median 42 days after surgery). In addition, DTI can only provide inferences about tract structure but not their functionality or of the cortical regions they connect; orthogonal methods such as functional MRI or direct electrical stimulation could provide this. Finally, we focused on the phases before and after surgery, but before any bias from chemoradiotherapy regimens. However, these require incorporating into future studies to fully understand the impact of each treatment phase and the natural history of the disease on social cognition.

## Conclusions

Emotion recognition deficits in glioblastoma patients undergoing resection surgery are associated with reduced FA in right hemisphere WM tracts; namely the IFOF, ILF, uncinate fasciculus and Anterior Thalamic Radiation. These deficits are already present in in a subgroup of patients before surgery and become more frequent after surgery. Our methodology demonstrates TBSS application for large, image distorting lesions and the results support the current mechanistic understanding of this particular deficit across pathologies. This research could aid patient consultation and inform future studies of social cognition rehabilitation for these patients.

## Supplementary material

Supplementary material is available at *Brain Communications* online.

## Funding

Cancer Research UK (RRZB/040 to R.S.) and the Royal College of Surgeons of England (RRAG/093); Fundatie van Renswoude (AV20180147 to A.D.); Cancer Research UK (CRUK/A19732 to C.L.); Medical Research Council (SUAG/049 and G101-400 to T.M.); National Institute for Health Research (NIHR), Career Development Fellowship for this research project (CDF-2018-11-ST2-003 to S.P.); NIHR Brain Injury MedTech Co-operative. This publication presents independent research funded by the National Institute for Health Research (NIHR). The views expressed are those of the author(s) and not necessarily those of the NHS, the NIHR or the Department of Health and Social Care.

## Competing interests

T.M. was involved in the development of OCS-BRIDGE and could potentially financially benefit in the future from its commercial release under rewards-to-inventor schemes of their respective institutions. The remaining authors report no competing interests.

## Supplementary Material

fcaa169_Supplementary_Data

## Abbreviations

DTI =: diffusion tensor imaging;
FA =: fractional anisotropy;
IFOF =: inferior fronto-occipital fasciculus;
ILF =: inferior longitudinal fasciculus;
SD: standard deviation;
TBSS =: tract-based spatial statistics;
UF =: uncinate fasciculus;
WM =: white matter
