# Supplementary material for: Glioblastoma surgery related emotion recognition deficits are associated with right cerebral hemisphere tract changes
Source: Brain Commun. 2020 Oct 12;2(2):fcaa169. doi: 10.1093/braincomms/fcaa169 (PMC7780443; doi:10.1093/braincomms/fcaa169)

Supplementary Material 1: MRI Acquisition

T2-weighted sequence [TR: 4840–5470ms; TE: 114ms; flip angle 150°; FOV: 220 × 165 mm; 23–26 sections; 0.5-mm section gap; voxel size, 0.7 × 0.7 × 5.0 mm; acquisition time, 93 seconds], T2-weighted fluid attenuated inversion recovery sequence (FLAIR) [TR: 7840–8420ms; TE: 95ms; TI: 2500ms; flip angle 150°; FOV: 250 × 200mm; 27 sections; 1-mm section gap; voxel size, 0.8 × 0.8 × 4.0mm; acquisition time, 268 seconds] a T1-weighted magnetization prepared rapid gradient echo (MPRAGE) sequence [TR: 2300ms, TE: 2.98 ms; flip angle 9°; FOV 256 x 240mm; voxel size 1.0 x 1.0 x 1.0 mm; acquisition times, 252 and 554 seconds] and a three-dimensional post-contrast T1-weighted MPRAGE sequence after the intravenous injection of 9ml gadolinium (Gadovist, Bayer Schering Pharma) [TR: 2300ms; TE: 2.98ms; TI: 900ms; flip angle, 9°; FOV: 256 × 240 mm; 176–208 sections; no section gap; voxel size, 1.0 × 1.0 × 1.0mm; acquisition times, 252 and 554 seconds]. For the Tract-Based Spatial Statistics (TBSS) analysis we acquired DTI scans before contrast enhanced imaging. We used a 2D echo-planar imaging (EPI), twice refocused spin-echo sequence, with 64 diffusion gradient directions with b = 1000 s/mm2 plus on non-weighted image with b=0 s/mm2 [TR: 8400 ms; TE: 90ms; FOV: 192 x 192 mm; 68 axial slices; voxel size 2.0 x 2.0 x 2.0 mm; GRAPPA acceleration factor 2; acquisition time, 570 seconds].

Supplementary Material 2: Preoperative Tumour Lesion Volume Correction

Preoperative 3D tumour volume was calculated from multimodal MRI sequences (T1, T1-post contrast, T2 and FLAIR) with use of the BraTumIA tool version 2.0 (software.istb.unibe.ch/registration/bratumia/). Steps included were (1) skull-stripping to generate a brain mask, (2) co-registration of the different MRI sequences to standard Montreal Neurological Institute (MNI) space to ensure voxel-to-voxel correspondence, (3) segmentation of the co-registered images into healthy tissue and tumour tissue based on combined classification and regularization, (4) production of a label map with quantitative information on the respective volume of enhancing tumour tissue, non-enhancing tumour tissue, necrotic tissue and oedema. There are suspicions that oedema may be associated with reduced cognitive functioning (Talacchi *et al.*, 2011; Campanella *et al.*, 2015; Farjam *et al.*, 2015). Therefore, all areas that were classified as non-healthy tissue were included as a covariate in the correlation analysis between change in emotion recognition score and change in FA values. In five cases visual inspection revealed suboptimal skull-stripping resulting into mis-segmentation of areas around the eye cavity as (non-)enhancing tumour tissue. For these patients we replaced the first step with FSL's Brain Extraction Tool (BET) to remove non-brain tissue (Smith, 2002). However, visual inspection still revealed suboptimal tumour lesion volume segmentation in three patients (Table 1). Accordingly, we replaced their tumour lesion volume estimates with the mean value of the group (normally distributed). We furthermore assumed that adjacent normal looking brain tissue was not resected during surgery in any of the patients. Accordingly, we classified preoperative brain tumour lesion volume to be similar to the postoperative cystic cavity.

References:

Campanella F, Fabbro F, Ius T, Shallice T, Skrap M. Acute effects of surgery on emotion and personality of brain tumor patients: Surgery impact, histological aspects, and recovery. Neuro Oncol 2015; 17: 1121–1131.

Farjam R, Pramanik P, Aryal MP, Srinivasan A, Chapman CH, Tsien CI, et al. A Radiation-Induced Hippocampal Vascular Injury Surrogate Marker Predicts Late Neurocognitive Dysfunction. Int J Radiat Oncol 2015; 93: 908–915.

Talacchi A, Santini B, Savazzi S, Gerosa M. Cognitive effects of tumour and surgical treatment in glioma patients. J Neurooncol 2011; 103: 541–549.

Supplementary Material 3: Scripts used in analysis pipeline

#Step1: DTI Parameters_Coregistration
Original script by Chao Li
Date modified script: 15 feb 2019, Aicha Dijkshoorn
Needed in order to run: Correct directory infrastructure: Patient001/DTI_preop directory (with in it DTI.nii and DTI.bvec and DTI.bval)
Function script: Create FA scan from the DTI.nii scan for each subject.
Running time: Approx 30-45 min/patient

% this script doesn't seem to run from a USB stick so run the script from fsluser/Documents etc.
% this script is written to ensure co-registration in the next step between DTI_preop/dti_FA.nii.gz and DTI_postop/dti_FA.nii.gz scans without having to rename each scan manually. If co-registration is not necessary the DTI_preop folder in each line has to be replaced by DTI (or scroll down to bottom script)

%%%%% preop FA %%%%%%%%%

Set the right path to the patient folder. To set up the loop make sure that the last directory of the path (in this case 'subjects') matches the word before the = and $ sign. Each patient starting with a *P (E.g. PatientXX) will be processed in a consecutive order ik you copy the script including the last line 'done'.
export subjects=/home/fsluser/Documents/CogENT_emotstudy/Aicha/preop/subjects/
cd $subjects

for patient in P*; do echo $patient;
export patient=/home/fsluser/Documents/CogENT_emotstudy/Aicha/preop/subjects/$patient/
cd $patient
=
Eddy current correction
echo "1/4 - Eddy current correction..."
eddy_correct DTI_preop/DTI.nii DTI_preop/data.nii.gz 0

Brain extraction - mask generation
echo "2/4 - BET brain extraction - generate binary brain mask image..."
fslroi DTI_preop/data.nii.gz DTI_preop/data_b0.nii.gz 0 1

Brain extraction - mask creation
bet DTI_preop/data_b0.nii.gz DTI_preop/data_brain_b0.nii.gz -f 0.05 -m

Rename the mask file
mv DTI_preop/data_brain_b0_mask.nii.gz DTI_preop/data_brain_mask.nii.gz

create DTI property values
echo "3/4 - DTIFit Reconstruct diffusion tensor..."

```
dtifit -k DTI_preop/data.nii.gz -m DTI_preop/data_brain_mask.nii.gz -r DTI_preop/DTI.bvec -b
DTI_preop/DTI.bval -o dti

#move DTI in folder
mv dti_FA.nii.gz DTI_preop/dti_FA.nii.gz
mv dti_MD.nii.gz DTI_preop/dti_MD.nii.gz
mv dti_L2.nii.gz DTI_preop/dti_L2.nii.gz
mv dti_L3.nii.gz DTI_preop/dti_L3.nii.gz
mv dti_L1.nii.gz DTI_preop/dti_L1.nii.gz
mv dti_V1.nii.gz DTI_preop/dti_V1.nii.gz
mv dti_V2.nii.gz DTI_preop/dti_V2.nii.gz
mv dti_V3.nii.gz DTI_preop/dti_V3.nii.gz
mv dti_MO.nii.gz DTI_preop/dti_MO.nii.gz
mv dti_S0.nii.gz DTI_preop/dti_S0.nii.gz

echo "4/4 - Calculate p and q maps ...  "
fslmaths DTI_preop/dti_MD.nii.gz -mul 1.732 DTI_preop/dti_p.nii.gz
fslmaths DTI_preop/dti_L1.nii.gz -sub DTI_preop/dti_MD.nii.gz -sqr
DTI_preop/dti_L1diff.nii.gz
fslmaths DTI_preop/dti_L2.nii.gz -sub DTI_preop/dti_MD.nii.gz -sqr
DTI_preop/dti_L2diff.nii.gz
fslmaths DTI_preop/dti_L3.nii.gz -sub DTI_preop/dti_MD.nii.gz -sqr
DTI_preop/dti_L3diff.nii.gz
fslmaths DTI_preop/dti_L1diff.nii.gz -add DTI_preop/dti_L2diff.nii.gz -add
DTI_preop/dti_L3diff.nii.gz -sqrt DTI_preop/dti_q.nii.gz

done
```

QC: open all DTI_FA scans in fsleyes/fslview_deprecated to check for artefacts
open all DTI_FA and V1 scans in fslview_deprecated to check whether the directional information of the tracks is correct

%%%%%% pre_RT FA %%%%%%%%%%%%

```
export subjects=/home/fsluser/Documents/CogENT_emotstudy/Aicha/postop/subjects/
cd $subjects

for patient in P*; do echo $patient;
export patient=/home/fsluser/Documents/CogENT_emotstudy/Aicha/postop/subjects/$patient/
cd $patient

# Eddy current correction
echo "1/4 - Eddy current correction..."
eddy_correct DTI_postop/DTI.nii DTI_postop/data.nii.gz 0

# Brain extraction - mask generation
```

```
echo "2/4 - BET brain extraction - generate binary brain mask image..."
fslroi DTI_postop/data.nii.gz DTI_postop/data_b0.nii.gz 0 1

# Brain extraction - mask creation
bet DTI_postop/data_b0.nii.gz DTI_postop/data_brain_b0.nii.gz -f 0.05 -m

# Rename the mask file
mv DTI_postop/data_brain_b0_mask.nii.gz DTI_postop/data_brain_mask.nii.gz

# create DTI property values
echo "3/4 - DTIFit Reconstruct diffusion tensor..."
dtifit -k DTI_postop/data.nii.gz -m DTI_postop/data_brain_mask.nii.gz -r DTI_postop/DTI.bvec
-b DTI_postop/DTI.bval -o dti

#move DTI in folder
mv dti_FA.nii.gz DTI_postop/dti_FA.nii.gz
mv dti_MD.nii.gz DTI_postop/dti_MD.nii.gz
mv dti_L2.nii.gz DTI_postop/dti_L2.nii.gz
mv dti_L3.nii.gz DTI_postop/dti_L3.nii.gz
mv dti_L1.nii.gz DTI_postop/dti_L1.nii.gz
mv dti_V1.nii.gz DTI_postop/dti_V1.nii.gz
mv dti_V2.nii.gz DTI_postop/dti_V2.nii.gz
mv dti_V3.nii.gz DTI_postopT/dti_V3.nii.gz
mv dti_MO.nii.gz DTI_postop/dti_MO.nii.gz
mv dti_S0.nii.gz DTI_postop/dti_S0.nii.gz

echo "4/4 - Calculate p and q maps ...  "
fslmaths DTI_postop/dti_MD.nii.gz -mul 1.732 DTI_postop/dti_p.nii.gz
fslmaths DTI_postop/dti_L1.nii.gz -sub DTI_postop/dti_MD.nii.gz -sqr
DTI_postop/dti_L1diff.nii.gz
fslmaths DTI_postop/dti_L2.nii.gz -sub DTI_postop/dti_MD.nii.gz -sqr
DTI_postop/dti_L2diff.nii.gz
fslmaths DTI_postop/dti_L3.nii.gz -sub DTI_postop/dti_MD.nii.gz -sqr
DTI_postop/dti_L3diff.nii.gz
fslmaths DTI_postop/dti_L1diff.nii.gz -add DTI_postop/dti_L2diff.nii.gz -add
DTI_postop/dti_L3diff.nii.gz -sqrt DTI_postop/dti_q.nii.gz

done
```

QC: open all DTI_FA scans in fsleyes/fslview_deprecated to check for artefacts
open all DTI_FA and V1 scans in fslview_deprecated to check whether the directional information of the tracks is correct

#Step2:CoregistrationFNIRT
Original script by Aicha Dijkshoorn
Date modified: 15 february 2019, Aicha Dijkshoorn
Needed in order to run: dti_FA scans at 2 different timepoints
Function script: Coregister FA-scan at timepoint 2 to the FA-scan at timepoint 1
Helpful source: https://fsl.fmrib.ox.ac.uk/fsl/fslwiki/FNIRT/UserGuide; supporting material word-file of van der Hoorn et al. (2016). NMR in Biomedicine
Running time: approx 15 min/patient

% after running script manually rename each subjects' preop FA file from subjects/Patient001/DTI_preop/dti_FA.nii.gz to subjects/TP1_Patient001_dti_FA.nii.gz
% after running script manually rename each subjects' postop FA file from subjects/Patient001/DTI_coreg/TP2_coreg_FA.nii.gz to TP2_Patient001_coreg_FA.nii.gz

Set the right path to the patient folder. To set up the loop make sure that the last directory of the path (in this case 'subjects') matches the word before the = and $ sign. Each patient starting with a *P (E.g. PatientXX) will be processed in a consecutive order ik you copy the script including the last line 'done'.
export subjects=/home/fsluser/Documents/CogENT_emotstudy/Aicha/subjects/
cd $subjects

for patient in P*; do echo $patient;
export patient=/home/fsluser/Documents/CogENT_emotstudy/Aicha/subjects/$patient/
cd $patient

linearly co-registrate the timepoint 2 FA image (postop) to timepoint 1 FA image (preop) to generate the transformation matrix (-omat)
flirt -ref DTI_preop/dti_FA.nii.gz -in DTI_postop/dti_FA.nii.gz -out flirt_coreg_postop_FA -omat coreg_postop_FA.mat -cost normmi -searchrx -90 90 -searchry -90 90 -searchrz -90 90 -dof 12 -interp trilinear

non-linearly co-registrate the timepoint 2 FA image (postop) to timepoint 1 FA image (preop) using the flirt transformation matrix (-omat = --aff)
fnirt --ref=DTI_preop/dti_FA.nii.gz --in=DTI_postop/dti_FA.nii.gz --aff=coreg_postop_FA.mat --cout=transform_postop --config=FA_2_FMRIB58_1mm.cnf

the generated output transformation file (--warp transform) is used to transform a timepoint 2 file that is in the same space as the timepoint 1 file
applywarp --ref=DTI_preop/dti_FA.nii.gz --in=DTI_postop/dti_FA.nii.gz --warp=transform_postop --out=TP2_coreg_FA

move the created files into DTI_coreg subdirectory and copy output in postop folder

mv TP2_coreg_FA.nii.gz DTI_coreg
mv coreg_postop_FA.mat DTI_coreg
mv flirt_coreg_postop_FA.nii.gz DTI_coreg

mv transform_postop.nii.gz DTI_coreg
cp DTI_preop/dti_FA.nii.gz DTI_coreg/preop_dti_FA.nii.gz

done

QC: check all coregistered scans in FSLoverlay by adjusting the luminance and see whether the central FA-tracts are well aligned

Step3:FASkeleton
Original script by Aicha Dijkshoorn
Date modified: 15 february 2019, Aicha Dijkshoorn
Needed in order to run: one folder (for instance /subjects) in which all the to-be-analysed (and if within subjects design also coregistered) FA-scans are stored in alphabetical order
Function script: Create mean FA skeleton for the entire group - Step 1-4 of the TBSS pipeline
Helpful source: https://fsl.fmrib.ox.ac.uk/fsl/fslwiki/TBSS/UserGuide
Running time: Approx 10 min per patient

% all dti_FA.nii.gz scans need to be (manually) named so it is clear from which patient and which timepoint (preop or postop) it is. Also, note that the alphabetical order of the patients will match the matrix design input, and therefore what will be considered group 1 (e.g. TP1*) and group 2 (e.g. TP2*)

path to directory
cd /home/fsluser/Documents/CogENT_emotstudy/Aicha/subjects/

#create a directory in which the TBSS output is stored
mkdir mytbss
mv TP1_*.nii.gz mytbss
mv TP2_*.nii.gz mytbss
cd mytbss

#erode the FA images slightly and zero the end slices
tbss_1_preproc *.nii.gz

#non-linearly all FA images to a 1x1x1mm standard space (approx 5min/subj). There are 3 co-registration options and we used co-registration to normal FMRIB58_FA space (-T). Rationale: option 2 -n (=most typical subject --> there is not a most typical GBM subject) or option 3 -t (=own target image, this would enable co-registration from timepoint 2 to timepoint 1 (which we already did), this does not allow comparison between subjects and we also do not have a disease specific GBM template yet))
tbss_2_reg -T

apply the nonlinear transformation to all subjects and bring them into standard space, thereby creating a standard-space mean FA and skeleton of the included subjects (rather than using the FMRIB58_FA standard skeleton)
tbss_3_postreg -S

tresholding the FA skeleton, thereby creating the projected skeletonised FA data. The default and recommended threshold is set at 0.2.
tbss_4_prestats 0.2

#view the mean skeleton
cd stats
fslview_deprecated all_FA -b 0,0.8 mean_FA_skeleton -b 0.2,0.8 -l Green

QC: Visually inspect slicerdir for obvious problems in scan quality
QC: Loop through the all_FA (vol 0 = subject 1, volume 1 = subject 2 etc.) to see whether the central tracts of the subjects are reasonably aligned with the mean_skeleton

#Step4:TBSSDesignMatrix
Original script by Aicha Dijkshoorn
Date modified: 15 february 2019, Aicha Dijkshoorn
Needed in order to run: mean FA skeleton as created by TBSS step 1-4
Function script: Create design matrix for the to-be-used study design (within-subjects OR between subjects)
Helpful sources: https://fsl.fmrib.ox.ac.uk/fsl/fslwiki/Randomise/UserGuide; https://fsl.fmrib.ox.ac.uk/fsl/fslwiki/FEAT/UserGuide#Paired_Two-Group_Difference_.28Two-Sample_Paired_T-Test.29
Time to create matrix: approx 5 min (within subjects)

% When you save the Glm-created-files the names of the folder in which it is saved (e.g. stats or mytbss directory) are automatically assigned to the .mat, .grp and .con file. To avoid confusion rename these files in a sensible way (e.g. surgery.mat or emotcorrection.mat)

#create a design.mat & design.con file with Randomise and/or FEAT guide
(1)insert Glm in the terminal (2) GLM Setup: select higher-level/ non-timeseries design; #inputs = number of patients x n timepoints (15 x 2 = 30) (3) Set up design matrix according to number of patients as described in the manuals (4) Rename the files stats.mat and stats.con (e.g. automatically name if you saved them in the stats directory) and rename them sensibly and drag / move them into the stats subdirectory

%structual FA -to- behavioural cognition analysis matrix (postup versus preop)%

#Glm design as in the structural analysis. Insert the emotion recognition task performance score at either EV1 (if no covariates included) or at EV2 (than to-be-controlled-covariate can be inserted under EV1)

Group (1st column)	EV1 (tumour volume)	EV2 (emotion_corr)
input 1: group 1	37	9
input 2: group 2	146	11
input 3: group 3	26	15
input 4: group 4	59	6

input 5: group 5	44	12
input 6: group 6	69	12
input 7: group 7	69	14
input 8: group 8	69	14
input 9: group 9	125	11
input 10: group 10	59	15
input 11: group 11	62	13
input 12: group 12	80	10
input 13: group13	36	14
input 14: group 14	89	12
input 15: group 15	69	14
input 16: group 1	37	6.3
input 17: group 2	146	9.5
input 18: group 3	26	11.6
input 19: group 4	59	8.5
input 20: group 5	44	11.6
input 21: group 6	69	10.6
input 22: group 7	69	11.6
input 23: group 8	69	13.8
input 24: group 9	125	9.5
input 25: group 10	59	14.8
input 26: group 11	62	11.6
input 27: group 12	80	7.4
input 28: group 13	36	14.8
input 29: group 14	89	7.4
input 30: group 15	69	12.7

Click on the tab Contrast & F-tests --> Contrasts: 2, F-tests:0. The 0 in the first tab means you control for the first column (EV1) as a covariate.
0 1
0 -1

#Glm design as in the structural analysis. Insert the face recognition task performance score at EV1 (no covariates included) or at EV2 (so the to-be-controlled-covariate can be inserted under EV1)

Group (1st column)	EV1 (tumour volume)	EV2 (faces)
input 1: group 1	37	5
input 2: group 2	146	6
input 3: group 3	26	5
input 4: group 4	59	5
input 5: group 5	44	6
input 6: group 6	69	6
input 7: group 7	69	6
input 8: group 8	69	6

input 9: group 9	125	4
input 10: group 10	59	4
input 11: group 11	62	6
input 12: group 12	80	5
input 13: group13	36	6
input 14: group 14	89	5
input 15: group 15	69	6
input 16: group 1	37	5
input 17: group 2	146	5
input 18: group 3	26	5
input 19: group 4	59	4
input 20: group 5	44	5
input 21: group 6	69	5
input 22: group 7	69	5
input 23: group 8	69	4
input 24: group 9	125	6
input 25: group 10	59	6
input 26: group 11	62	4
input 27: group 12	80	4
input 28: group 13	36	6
input 29: group 14	89	4
input 30: group 15	69	4

Click on the tab Contrast & F-tests --> Contrasts: 2, F-tests:0. The 0 in the first tab means you control for the first column (EV1) as a covariate.
0 1
0 -1

#Glm design as in the structural analysis. Insert the face recogition task performance score at EV1 (no covariates included) or at EV2 (so the to-be-controlled-covariate can be inserted under EV1)

Group (1st column)	EV1 (tumour volume)	EV2 (huts)
input 1: group 1	37	6
input 2: group 2	146	6
input 3: group 3	26	6
input 4: group 4	59	5
input 5: group 5	44	5
input 6: group 6	69	6
input 7: group 7	69	5
input 8: group 8	69	2
input 9: group 9	125	4
input 10: group 10	59	5
input 11: group 11	62	5
input 12: group 12	80	5

input 13: group13	36	6
input 14: group 14	89	5
input 15: group 15	69	3
input 16: group 1	37	5
input 17: group 2	146	4
input 18: group 3	26	5
input 19: group 4	59	5
input 20: group 5	44	5
input 21: group 6	69	4
input 22: group 7	69	5
input 23: group 8	69	4
input 24: group 9	125	5
input 25: group 10	59	5
input 26: group 11	62	4
input 27: group 12	80	5
input 28: group 13	36	6
input 29: group 14	89	4
input 30: group 15	69	4

Click on the tab Contrast & F-tests --> Contrasts: 2, F-tests:0. The 0 in the first tab means you control for the first column (EV1) as a covariate.
0 1
0 -1

#Glm design as in the structural analysis. Insert the face recognition task performance score at EV1 (no covariates included) or at EV2 (so the to-be-controlled-covariate can be inserted under EV1)

Group (1st column)	EV1 (tumour volume)	EV2 (line bisection)
input 1: group 1	37	-0.8
input 2: group 2	146	2.0
input 3: group 3	26	0.6
input 4: group 4	59	3.8
input 5: group 5	44	4.0
input 6: group 6	69	-2.5
input 7: group 7	69	10.6
input 8: group 8	69	-1.1
input 9: group 9	125	-1.6
input 10: group 10	59	0
input 11: group 11	62	0.9
input 12: group 12	80	0.4
input 13: group13	36	1.0
input 14: group 14	89	1.7
input 15: group 15	69	1.6
input 16: group 1	37	-3.8

input 17: group 2	146	0.4
input 18: group 3	26	7.2
input 19: group 4	59	15.2
input 20: group 5	44	-0.4
input 21: group 6	69	-3.6
input 22: group 7	69	16.3
input 23: group 8	69	1.6
input 24: group 9	125	1.0
input 25: group 10	59	10.8
input 26: group 11	62	0.4
input 27: group 12	80	4.0
input 28: group 13	36	-2.6
input 29: group 14	89	0.4
input 30: group 15	69	0.4

Click on the tab Contrast & F-tests --> Contrasts: 2, F-tests:0. The 0 in the first tab means you control for the first column (EV1) as a covariate.
0 1
0 -1

#Step5VoxelwiseStatistics
Original script by Aicha Dijkshoorn
Date modified: 15 february 2019, Aicha Dijkshoorn
Needed in order to run: mean FA skeleton as created by TBSS pipeline and the generated matrix files in case of within-subject analysis with emot_corr_15pt.con, emot_corr_15pt.grp, emot_corr_15pt.mat, emot_corr_15pt.fsf - or any variation on the chosen name
Function script: Conduct structural voxelwise analysis or postop versus preop FA-to- postop versus preop cognition voxelwise analysis for the entire group
Helpful sources: https://fsl.fmrib.ox.ac.uk/fsl/fslwiki/TBSS/UserGuide (=structural part), https://web.stanford.edu/group/vista/cgi-bin/wiki/index.php/MrVista_TBSS (=structural correlated to social cognition)
Time needed to run: 3-4 hours (depending on the amount of permutations which should be 5.000 or 10.000)

% MNI brain template for display purposes can be found in the following subdirectory: $FSLDIR/data/standard/MNI152_T1_1mm (you can copy this into the /stats folder)

check in which order the groupfiles are
cd FA
imglob *_FA.*
cd ../

% structural FA-to-behavioural cognition task scores within-subjects GLM design voxelwise statistics %

% IMPORTANT NOTE: there were four patients with suspicion of tumour segmentation errors around the eye cavity (patient009, patient013, patient015, patient016) after application of bet in fsl patient009 was segmented properly. The tumour volume of patient013, patient015 and patient16 were replaced with the mean tumour lesion volume (69cm3) of the included patients.

correlation to behavioral emotion task measures (information retrieved from: https://web.stanford.edu/group/vista/cgi-bin/wiki/index.php/MrVista_TBSS) - the names are manually created in step 4: Glm matrix design
randomise -i all_FA_skeletonised -o emot_corr_tumvol_mean_15pt -m mean_FA_skeleton_mask -d emot_corr_tumvol_mean_15pt.mat -t emot_corr_tumvol_mean_15pt.con -e emot_corr_tumvol_mean_15pt.grp -n 10000 --T2 -D
randomise -i all_FA_skeletonised -o face_tumvol_mean_15pt -m mean_FA_skeleton_mask -d face_tumvol_mean_15pt.mat -t face_tumvol_mean_15pt.con -e face_tumvol_mean_15pt.grp -n 10000 --T2 -D
randomise -i all_FA_skeletonised -o huts_tumvol_mean_15pt -m mean_FA_skeleton_mask -d huts_tumvol_mean_15pt.mat -t huts_tumvol_mean_15pt.con -e huts_tumvol_mean_15pt.grp -n 10000 --T2 -D
randomise -i all_FA_skeletonised -o line_tumvol_mean_15pt -m mean_FA_skeleton_mask -d line_tumvol_mean_15pt.mat -t line_tumvol_mean_15pt.con -e line_tumvol_mean_15pt.grp -n 10000 --T2 -D

#display positive (_tstat1) and negative correlations(_tstat2) between skeletonized FA of all subjects and ajusted emotion recognition and tumourvolume correction
fslview_deprecated $FSLDIR/data/standard/MNI152_T1_1mm mean_FA_skeleton -l Green -b 0.2,0.7 emot_corr_tumvol_mean_15pt_tfce_corrp_tstat1 -l Red-Yellow -b 0.95,1
fslview_deprecated $FSLDIR/data/standard/MNI152_T1_1mm mean_FA_skeleton -l Green -b 0.2,0.7 emot_corr_tumvol_mean_15pt_tfce_corrp_tstat2 -l Red-Yellow -b 0.95,1

displaying TBSS results (corrected, thickened tracts) - contrast 1 > contrast 2
tbss_fill emot_corr_tumvol_mean_15pt_tfce_corrp_tstat1 0.95 mean_FA tbss_fill_emot_corr_tumvol_mean_15pt
fslview_deprecated mean_FA -b 0,0.6 mean_FA_skeleton -l Green -b 0.2,0.7 tbss_fill_emot_corr_tumvol_mean_15pt -l Red-Yellow

#display positive (_tstat1) and negative correlations(_tstat2) between skeletonized FA of all subjects and uncorrected face recognition
fslview_deprecated $FSLDIR/data/standard/MNI152_T1_1mm mean_FA_skeleton -l Green -b 0.2,0.7 face_tumvol_mean_15pt_tfce_corrp_tstat1 -l Red-Yellow -b 0.95,1
fslview_deprecated $FSLDIR/data/standard/MNI152_T1_1mm mean_FA_skeleton -l Green -b 0.2,0.7 face_tumvol_mean_15pt_tfce_corrp_tstat2 -l Red-Yellow -b 0.95,1

displaying TBSS results (corrected, thickened tracts) - contrast 1 > contrast 2
tbss_fill face_tumvol_mean_15pt_tfce_corrp_tstat1 0.95 mean_FA tbss_fill_face_tumvol_mean_15pt
fslview_deprecated mean_FA -b 0,0.6 mean_FA_skeleton -l Green -b 0.2,0.7 tbss_fill_face_tumvol_mean_15pt -l Red-Yellow

#display positive (_tstat1) and negative correlations(_tstat2) between skeletonized FA of all subjects and object(huts) recognition
fslview_deprecated $FSLDIR/data/standard/MNI152_T1_1mm mean_FA_skeleton -l Green -b 0.2,0.7 huts_tumvol_mean_15pt_tfce_corrp_tstat1 -l Red-Yellow -b 0.95,1
fslview_deprecated $FSLDIR/data/standard/MNI152_T1_1mm mean_FA_skeleton -l Green -b 0.2,0.7 huts_tumvol_mean_15pt_tfce_corrp_tstat2 -l Red-Yellow -b 0.95,1

displaying TBSS results (corrected, thickened tracts) - contrast 1 > contrast 2
tbss_fill huts_tumvol_mean_15pt_tfce_corrp_tstat1 0.95 mean_FA tbss_fill_huts_tumvol_mean_15pt
fslview_deprecated mean_FA -b 0,0.6 mean_FA_skeleton -l Green -b 0.2,0.7 tbss_fill_huts_tumvol_mean_15pt -l Red-Yellow

#display positive (_tstat1) and negative correlations(_tstat2) between skeletonized FA of all subjects and object (huts) recognition
fslview_deprecated $FSLDIR/data/standard/MNI152_T1_1mm mean_FA_skeleton -l Green -b 0.2,0.7 line_tumvol_mean_15pt_tfce_corrp_tstat1 -l Red-Yellow -b 0.95,1
fslview_deprecated $FSLDIR/data/standard/MNI152_T1_1mm mean_FA_skeleton -l Green -b 0.2,0.7 line_tumvol_mean_15pt_tfce_corrp_tstat2 -l Red-Yellow -b 0.95,1

displaying TBSS results (corrected, thickened tracts) - contrast 1 > contrast 2
tbss_fill line_tumvol_mean_15pt_tfce_corrp_tstat1 0.95 mean_FA tbss_fill_line_tumvol_mean_15pt
fslview_deprecated mean_FA -b 0,0.6 mean_FA_skeleton -l Green -b 0.2,0.7 tbss_fill_line_tumvol_mean_15pt -l Red-Yellow

% how to display the sig. pictures in FSLeyes?
(0) type fsleyes in the terminal
(1) file --> open $FSLDIR/data/standard/MNI152_T1_1mm (or copy this into the stats folder)
(2) file --> open stats/mean_FA_skeleton --> min = 0.2, max =0.7, colour = green
(3) file --> open stats/*_corrp_tstats1.nii.gz --> min = 0.95, max = 1.0, colour = red – yellow

#Step6ClusterTractExtraction
Original script by Aicha Dijkshoorn
Date modified: 15 february 2019, Aicha Dijkshoorn
Needed in order to run: voxelwise statistic outcome (_tfce_corrp_tstat1.nii.gz) files
Function script: Label significant clusters with JHU White-Matter Tractography atas
Helpful sources: https://fsl.fmrib.ox.ac.uk/fsl/fslwiki/Cluster; https://www.researchgate.net/post/How_can_I_extract_significant_clusters_their_sizes_MNI_coordinates_and_corresponding_FA_values_after_randomise_in_TBSS_analysis2,

% the cluster_*_95.output shows a table with the sig. clusters with MNI XYZ coordinates
% to automatically localise in which tracts these sig. clusters are localised you have two options for the atlasq ohi option (1) create a mask of the sig. cluster (it is perhaps possible with longitudinal data, but needs more code and manual steps) (2) insert the MNI xyz coordinates of

maximum intensity voxel (3) insert the MNI xyz coordinate of the Centre of Gravity for the cluster (e.g. weighted average of coordinates by intensities within the cluster)

extract the significant tracts - behavioral statistics clusters: cluster size, coordinates and Z-value.
cluster -i emot_tumvol_mean_15pt_tfce_corrp_tstat1 -o cluster_index_emot_tumvol_mean_15pt -t 0.95 --mm > cluster_index_emot_tumvol_mean_15pt_.05.output
cluster -i face_tumvol_mean_15pt_tfce_corrp_tstat1 -o cluster_index_face_tumvol_15pt -t 0.95 --mm > cluster_face_tumvol_15pt_.05.output
cluster -i huts_tumvol_mean_15pt_tfce_corrp_tstat1 -o cluster_index_huts_tumvol_15pt -t 0.95 --mm > cluster_huts_tumvol_15pt_.05.output
cluster -i line_tumvol_mean_15pt_tfce_corrp_tstat1 -o cluster_index_line_tumvol_15pt -t 0.95 --mm > cluster_line_tumvol_15pt_.05.output

#cluster localization emotional score (p<.05) via manually inserted max XYZ coordinate from the cluster_index_emot_tumvol_mean_15pt_.05.output textfile
atlasq ohi -a "JHU White-Matter Tractography Atlas" -c 38,-10,-16 ILOF-R, ILF-R, UF-R, ATR-R

#cluster localization emotional score (p<.05) via manually inserted CoG XYZ coordinate (last 3 columns) from the cluster_index_emot_tumvol_mean_15pt_.05.output textfile
atlasq ohi -a "JHU White-Matter Tractography Atlas" -c 37.1, -23.5, -13.6 IFOF-R, ILF-R

#cluster localization face score after tumvolume correction (p<.05) via manually inserted max XYZ coordinate from the cluster_face_tumvol_15pt_.05.output textfile
ns

#cluster localization face score after tumour volume correction (p<.05) via manually inserted CoG XYZ coordinate from the cluster_face_tumvol_15pt_.05.output textfile
ns

#cluster localization huts score after tumour volume correction (p<.05) via manually inserted max XYZ coordinate from the cluster_huts_t1_95.output textfile
ns

#cluster localization huts score after tumour volume correction (p<.05) via manually inserted CoG XYZ coordinate from the cluster_huts_t1_95.output textfile
ns

#cluster localization line score after tumour volume correction(p<.05) via manually inserted max XYZ coordinate from the cluster_line_tumvol_15pt_.05.output textfile
ns

#cluster localization line score after tumour volume correction (p<.05) via manually inserted CoG XYZ coordinate from the cluster_line_tumvol_15pt_.05.output textfile
Ns

Supplementary Table 1: Comparison Analysis Included and Excluded Patients

	**Included patients** (***N*=15*)***	**Excluded patients** (***N*=8)**	***Sig.***
Demographics			
Age, mean±SD	57.6 (10.1)	62.4 (6.3)	.239
Sex, no females (%)	5 (33.3)	3 (37.5)	.842
Years of education, mean (±SD)	17.1 (5.0)	14.1 (3.5)	.142
Tumour characteristics			
Tumour lateralization, no left (%)	8 (53.3)	6 (75)	.311
Tumour location			.085
Frontal, no (%)	4 (26.7)	0 (0)	-
Temporal, no (%)	7 (46.7)	5 (62.5)	-
Parietal, no (%)	0 (0)	2 (25.0)	-
Occipital, no (%)	4 (26.7)	1 (12.5)	-
Tumour volume, mean (±SD)	70.3 (32.2)*	81.0 (33.8)	.421
IDH1, wildtype no (%)	14 (93.3)	8 (100)	.455
Ki67, mean±SD	29.0 (12.6)	30.1 (21.4)	.884
MGMT, methylated (%)	8 (53.3)	2 (28.6)**	.277
EOR > 90%, no (%)	15 (100)	8 (100)	N/A
Cognitive characteristics			
Emotion recognition score, mean (±SD)	12.1 (2.5)	12.0 (1.6)	.892
Face recognition score, median (range)	6.0 (4 - 6)	5.5 (4 – 6)***	.935****
Object recognition score, median (range)	5.0 (2 - 6)	4.5 (2 – 6)***	.249****
Line bisection score, mean (±SD)	1.4 (3.1)	-3.4 (3.8)	.003
GAD, mean (±SD)	8.3 (7.0)	9.0 (6.1)	.804
PHQ, mean (±SD)	7.3 (6.9)	10.2 (6.6)	.324

Continuous data are depicted in mean + standard deviation in case data are normally distributed or median (range) otherwise. Binary or nominal outcomes measures are depicted as number (%). To compare whether patients that remained eligible in the study differed from patients were excluded we used Independent sample t-test for continuous data and the Chi Square test for binary or nominal data. EOR = extent of resection, GAD = general anxiety disorder questionnaire, IDH1 = Isocitrate dehydrogenase 1 enzyme, Ki67 = Antigen identified my monoclonal antibody KI-67MGMT = O(6)-Methylguanine-DNA-methyltransferase, N/A = Not applicable, No = number, Perc = perception, PHQ = patient health questionnaire, Sig. = significance, SD = standard deviation

* We observed tumour lesion segmentation errors in four patients. For one patient removal of non-brain tissue was performed with BET and for three patients the tumour lesion volume was replaced with the mean value of the included patients (normally distributed).

** For one patient we were unable to find the methylation status for the promotor of the MGMT-gene.

*** Missing data from one patient that did not carry out the FAB subtest

**** *p*- value equal variances not assumed

Supplementary Table 2: Raw Cognitive Tests Data and Questionnaires Data

	Emotion recognition score		Face recognition score		Object recognition score		Line bisection score		GAD-7 score		PHQ9 score	
	**Preop**	**Postop***	**Preop**	**Postop**	**Preop**	**Postop**	**Preop**	**Postop**	**Preop**	**Postop**	**Preop**	**Postop**
Pt001	9	6.3	5	5	6	5	-0.8	-3.8	1	0	1	0
Pt002	11	9.5	6	5	6	4	2.0	0.4	8	4	9	3
Pt003	15	11.6	5	5	6	5	.6	7.2	8	2	7	3
Pt004	6	8.5	5	4	5	5	3.8	15.2	2	1	3	2
Pt005	12	11.6	6	5	5	5	4.0	-0.4	5	0	8	0
Pt006	12	10.6	6	5	6	4	-2.5	-3.6	0	0	0	0
Pt007	14	11.6	6	5	5	5	10.6	16.3	20	0	1	1
Pt008	14	13.8	6	4	2	4	-1.1	1.6	3	3	2	1
Pt009	11	9.5	4	6	4	5	-1.6	1.0	16	16	23	15
Pt010	15	14.8	4	6	5	5	0.0	10.8	0	0	2	1
Pt011	13	11.6	6	4	5	4	0.9	0,4	5	2	8	5
Pt012	10	7.4	5	4	5	5	0.4	4.0	10	6	9	6
Pt013	14	14.8	6	6	6	6	1.0	-2.6	12	1	6	9
Pt014	12	7.4	5	4	5	4	1.7	0.4	21	18	21	23
Pt015	14	12.7	6	4	3	4	1.6	0.4	13	11	10	15

GAD-7 = General Anxiety Disorder questionnaire, PHQ9 = Patient Health Questionnaire, Postop = postoperative, Preop = preoperative,
*Corrected for repeated testing effects (see supplementary table 2)

Supplementary Table 3: Raw Cognitive Test Data of Normative Control Group

	Emotion recognition score	
	Version A	Version B (Parallel Re-Test)
CON001	14	14
CON002	16	14
CON003	16	14
CON004	16	11
CON005	12	12
CON006	14	12
CON007	12	13
CON008	11	10
CON009	15	15
CON010	14	13
CON011	12	14
CON012	16	15
CON013	12	16
CON014	12	14
CON015	14	12
CON016	14	15
CON017	16	9
CON018	18	16
CON019	16	13
CON020	15	13
CON021	16	12
CON022	11	14
CON023	14	14
CON024	14	16
CON025	14	13
CON026	16	16
CON027	12	11
CON028	15	10
CON029	15	17
Deficit cut-off (2SD)	<11	<10

Raw cognitive data of the parallel test versions in 29 normative controls. These test result suggest that version B is slightly more difficult. Corrections calculated from these changes between Version A and Version B.

Supplementary Table 4: TBSS Tract Co-ordinates Emotion Recognition and FA-tract Deterioration

**Regions (Max XYZ)**	**Max MNI Coordinates (XYZ)**			**Regions (CoG XYZ)**	**CoG MNI Coordinates (XYZ)**			***N* of voxels**	***p*-value**
IFOF-R<br>ILF-R<br>UF-R<br>ATR-R	38	-10	-16	IFOF-R<br>ILF-R	37.1	-23.5	-13.6	2871	.014

Coordinates for the peak values (Max MNI) and the weighted average of the intensity values (CoG MNI) are displayed. The data are corrected for Family-wise error (FWE). ATR-R: Right Anterior Thalamic Radiation, CoG: Centre of Gravity value, IFOF-R: Right Inferior Fronto Occipital Fasciculus, ILF-R: Right Inferior Longitudinal Fasciculus, Max: Maximum intensity value, MNI: Montreal Neuro imaging standard space, UF-R: Right Uncinate Fasciculus

Supplementary Figure 1: TBSS correlation Analysis FA-tract Changes and Cognitive Test Performance on Multiplanar Significance Map

Supplementary Fig. 1. Multiplanar presentation of the correlation analysis after preoperative tumour lesion correction between longitudinal changes on performance on emotion recognition task performance and longitudinal FA deterioration. There is a significant positive correlation (*p*<.05) between decreased performance on the emotion recognition task and decreased FA values (TFCE-corrected) in the right ILF, the right IFOF and the right Cingulum.

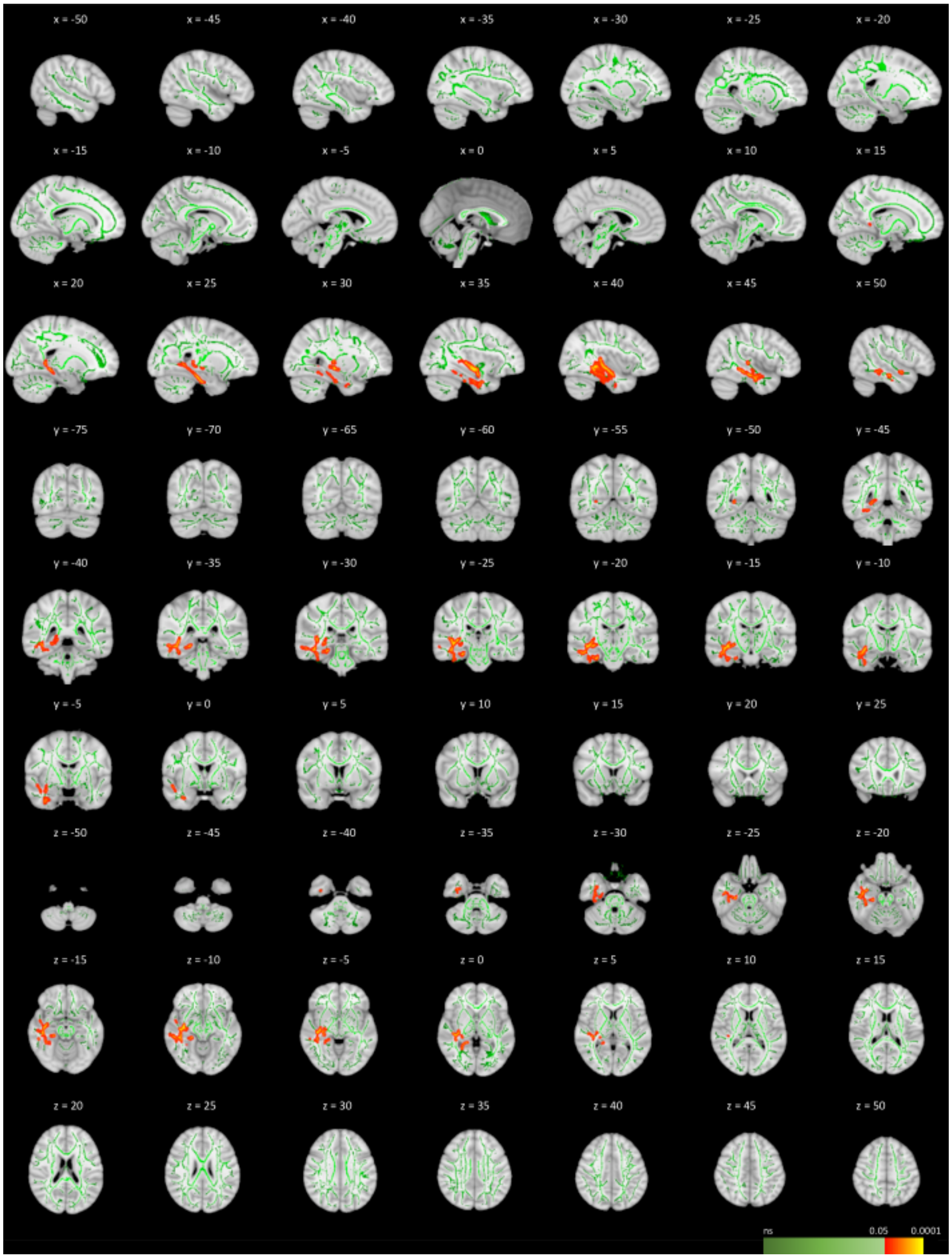

Supplement: fcaa169_Supplementary_Data [file fcaa169_supplementary_data.pdf]
